# The pancreatic surgery registry (StuDoQ|Pancreas) of the German Society for General and Visceral Surgery (DGAV) – presentation and systematic quality evaluation

**DOI:** 10.1186/s13063-017-1911-x

**Published:** 2017-04-05

**Authors:** Ulrich F. Wellner, Carsten Klinger, Kai Lehmann, Heinz Buhr, Edmund Neugebauer, Tobias Keck

**Affiliations:** 1 German Society for General and Visceral Surgery (DGAV), Haus der Bundespressekonferenz, Schiffbauerdamm 40, Mieteinheit 3.200, 10117 Berlin, Germany; 2 German Society for General and Visceral Surgery (DGAV), StuDoQ Registry, Berlin, Germany; 3 German Society for General and Visceral Surgery (DGAV), Integrated National Study Group Pancreas, Berlin, Germany; 4 Klinik für Chirurgie, UKSH Campus Lübeck, Lübeck, Germany; 5 Private Universität Witten/Herdecke, Witten, Germany

**Keywords:** Pancreatic surgery, Clinical registry, Quality assessment, StuDoQ|Pancreas

## Abstract

**Background:**

Pancreatic resections are among the most complex procedures in visceral surgery. While mortality has decreased substantially over the past decades, morbidity remains high. The volume-outcome correlation in pancreatic surgery is among the strongest in the field of surgery. The German Society for General and Visceral Surgery (DGAV) established a national registry for quality control, risk assessment and outcomes research in pancreatic surgery in Germany (DGAV SuDoQ|Pancreas).

**Methods:**

Here, we present the aims and scope of the DGAV StuDoQ|Pancreas Registry. A systematic assessment of registry quality is performed based on the recommendations of the German network for outcomes research (DNVF).

**Results:**

The registry quality was assessed by consensus criteria of the DNVF in regard to the domains Systematics and Appropriateness, Standardization, Validity of the sampling procedure, Validity of data collection, Validity of statistical analysis and reports, and General demands for registry quality. In summary, DGAV StuDoQ|Pancreas meets most of the criteria of a high-quality clinical registry.

**Conclusion:**

The DGAV StuDoQ|Pancreas provides a valuable platform for quality assessment, outcomes research as well as randomized registry trials in pancreatic surgery.

**Electronic supplementary material:**

The online version of this article (doi:10.1186/s13063-017-1911-x) contains supplementary material, which is available to authorized users.

## Background

Pancreatic surgery has become widely established in the Western world, and is usually practiced by specially trained surgeons in various settings, ranging from community hospitals to high-volume academic centers [1–5]. High perioperative morbidity and health care costs as well as a strong volume-outcome relationship have been demonstrated for pancreatic surgery [6–11], leading to an increased demand for quality assessment and assurance.

So far, national practice guidelines including quality benchmarks have been developed mostly on the basis of data reported from specialized centers, as well as from randomized controlled trials (RCTs) [12–14]. The usability of health care insurance data (diagnostic-related categories, DRG) to classify pancreatic surgery in Germany has been analyzed for quality assessment and assurance purposes by recent publications from Germany [15]. These data demonstrate a relevant morbidity and mortality from pancreatic surgery overall and that a detailed analysis and establishment of risk factors for pancreatic surgery are clearly warranted. However, it also became obvious that nonadjusted administrative routine data have significant shortcomings in this matter [16]. An alternative to administrative data is data from clinical registries.

“Real-world data” derived from high-quality registries in general are able (1) to close the information gap between the virtual world of RCTs and daily practice and (2) to follow-up the effect of guidelines on the quality of care. In responsibility to the growing academic and political interest in patient safety and quality assurance, the German Society for General and Visceral Surgery (Deutsche Gesellschaft für Allgemein- und Viszeralchirurgie, DGAV) decided to establish a so-called StuDoQ|Pancreas Registry for pancreatic surgery. The registry enables surgeons to assess risk factors for impaired perioperative outcomes as well as guideline adherence on the institutional level, in comparison with the national average. Benchmarking with risk/case-mix correction is one of the specific aims. Steps towards this aim are: (1) to define the best quality indicators for the registry, and (2) to correct these quality measures by risk profile. The first step has already been completed (DGAV quality commission 2017, manuscript in preparation), while the second step is being undertaken. Furthermore, StuDoQ|Pancreas provides a framework for outcomes research and future registry-based randomized controlled trials (RRCTs) in pancreatic surgery.

To fulfill these various demands, the registry itself must have a high quality. The German Network Health Services Research (www.dnvf.de) developed a memorandum with criteria that allow the assessment and evaluation of the quality of a registry [17]. These criteria were applied to assess the DGAV StuDoQ|Pancreas Registry.

## Methods

### The DGAV StuDoQ|Pancreas Registry

The registry was developed as a tool for risk-adjusted quality assessment in pancreatic surgery. Furthermore, it is intended to provide a framework suited for scientific analysis in the scope of surgical and outcomes research. With regard to these aims, the following aspects were elaborated by a development panel of surgeons with expertise in pancreatic surgery, one surgeon with registry expertise (KL) and one information scientist (CK) and reviewed by a senior expert panel. The surgeons of the panel were derived from five German high-volume academic hospitals and the DGAV. Relevant outcomes and quality indicators were chosen by expert consensus from the German S3 guideline for the treatment of pancreatic cancer [13], the guidelines for pancreatic cancer center certification of the German Cancer Society (DKG, www.krebsgesellschaft.de) and current reported randomized controlled trials (RCTs) in pancreatic surgery. Definitions of the International Study Group for Pancreatic Surgery (ISGPS) were applied where possible. Relevant risk factors were chosen by expert consensus after a review of the current medical literature, with emphasis on reported RCTs in pancreatic surgery and the American College of Surgeons National Surgical Quality Improvement Program (ACS-NSQIP).

The registry was implemented as an SQL database with browser-based data entry. Data from participating centers are prospectively entered in pseudonymized form using a browser-based tool which undergoes automatic plausibility control. Validation by cross-checking with institutional medical controlling data is part of the annual pancreatic cancer center certification process and was considered necessary for random sample data monitoring. The informed consent and data safety concept was approved by the Society for Technology, Methods and Infrastructure for Networked Medical Research (http://www.tmf-ev.de) and publication guidelines were established by the DGAV (http://www.dgav.de/studoq/datenschutzkonzept-und-publikationsrichtlinien.html).

### Evaluation of the registry quality

Registries in health services research vary in their aims and research questions as well as in their designs, methods of data collection, and statistical analyses. Standard criteria used for evaluation of quality of clinical registries have been developed and consented by the German Network Health Services Research (www.dnvf.de) [17]. The focus during establishment of DGAV StuDoQ|Pancreas was on the scientific validity of risk factors and outcome parameters, as well as on practicability. The stimulus for application of the DNVF Checklist was given by one of the coauthors with special expertise on outcomes research (EN). The Checklist for assessment of the quality of the registry is provided in Table 1. Two persons (CK, UW) assessed the registry accordingly followed by a third person (EN) for validation. Results are provided as text.

**Table 1:** The German Network Health Services Research (www.dnvf.de) criteria for assessment of registry quality. For details see [17]

Domain	Item
1. Systematics and Appropriateness	Registry protocol
	Aims and expected benefits
	Registry organization
	Patient rights and data safety
	Registry design
	Data acquisition and processing
	Data analyses, reporting and publication
	Steering Committee
2. Standardization	Definitions and standard operating procedures
	Training for data entry and verification
3. Validity of the sampling procedure	Definition of inclusion and exclusion criteria
	Completeness and representativity of data
	Consideration of sample size and effect
	Consideration of confounders and biases
4. Validity of data collection	Assessment of data: completeness, plausibility, distribution, Concordance
	Handling of missing data, follow-up and dropout
	Unique identifiers and risk of duplicate data entry
	Monitoring and audits
5. Validity of statistical analyses and reports	Patient/data flow scheme
	Handling of missing data
	Assessment of baseline data and outcome measures, Evaluation of balance in comparative analyses
	Assessment of precision measures
	Methods against bias and confounders
	Adjustment for multiple inference testing
	Assessment of relative and absolute effects, adjusted and Unadjusted results
	Multivariate modeling for complex questions
	Consideration of timeline in longitudinal data
	Control of cluster effects
	Validity of statistical analyses and reports
6. General demands for registry quality	Transparency towards limitations
	Acceptance among patients and institutions
	Transparency and scientific independence
	Flexibility and adaptability
	Topicality

## Results and discussion

The evaluation presented here follows the structure suggested by the DNVF memorandum on clinical registries (Table 1) [17].

### Domain: Systematics and Appropriateness

#### Registry protocol

A formal protocol detailing aims and regulations for data safety and publication has been established by the DGAV and can be accessed publicly at http://www.dgav.de/studoq/datenschutzkonzept-und-publikationsrichtlinien.html. This manuscript also represents an abridged form to fulfill standards for documentation of registries.

#### Aims and expected benefits

The registry serves several aims. The first aim is to provide a tool for risk-adapted quality management in pancreatic surgery. Hereby, it is intended to close the gap between unadjusted routine data and registry data. A second aim is to assess guideline adherence in the treatment of pancreatic cancer, as elaborated by the German Cancer Society (DKG, www.krebsgesellschaft.de) [13], to be used for certification purpose in the annual recertification of pancreatic cancer centers in Germany. The registry also allows assessment of certification requirements of centers for pancreatic surgery, as accredited by the DGAV in Germany. Third, the registry provides a basis for outcomes research in the form of retrospective studies. Fourth, there is a potential for further development as a framework for prospective randomized registry trials [18, 19] and biomaterial studies.

#### Registry organization

The DGAV acts according to its constitution as a professional association of general and visceral surgeons in Germany. DGAV StuDoQ|Pancreas is an organ-specific part of the DGAV StuDoQ registry, hosted by the DGAV and developed in close association with the German Chapter of the International Hepato-pancreato-biliary Association (IHPBA) known as CALGP. The Executive Board of the DGAV StuDoQ Registry is formed according to the registry constitution (available at http://www.dgav.de/studoq/datenschutzkonzept-und-publikationsrichtlinien.html). The tasks comprise establishment, regulation, control, and further development of the legal, medical, and scientific aspects of the registry. Steering Committees are established for all organ-specific parts of the DGAV StuDoQ Registry. The DGAV employs professional personnel for further development and maintenance of the registry at the level of IT implementation. The DGAV StuDoQ database is hosted by the DGAV and physically located in the DGAV headquarters in Berlin, Germany, thereby assuring independent data management.

#### Patient rights and data safety

National regulations require consent for pseudonymized data collection in registries. Apart from quality control, the registry is also intended for scientific analysis and outcomes research. Written informed consent for participation in the registry is obtained from all patients. Patient data are entered and stored in pseudonymized form. The Informed Consent Form and data safety concept have been approved by the Society for Technology, Methods and Infrastructure for Networked Medical Research of Germany (http://www.tmf-ev.de). A Data Safety Manual detailing the implementation of consenting and data safety at the institutional level has been published by the DGAV and can be accessed at the DGAV website (www.dgav.de). For possible randomized registry trials, additional informed consent is needed.

#### Registry design

The DGAV StuDoQ|Pancreas Registry is designed as a prospectively maintained multicenter database with web-based data entry. Observed units are patients undergoing pancreatic surgery (various procedures, for details see inclusion and exclusion criteria below) in the participating institutions. Parameters assessed are defined by the browser-based electronic Case Report Form (eCRF; for a list of assessed parameters see Additional file 1).

#### Data acquisition and processing

Data acquisition is performed by a browser-based eCRF. Entered data undergo immediate automatic plausibility check with automatic query generation. The aspects of plausibility control were elaborated during the process of registry implementation in the form of expert consensus.

#### Data analyses, reporting and publication

Analyses of the registry data are possible in different ways. First, annual statistics are calculated and reported to the participating institutions as quality reports. These comprise descriptive statistics of baseline parameters, risk factors, operation details, perioperative outcome, oncologic quality indicators, and follow-up. Further, operative risk and risk-adapted outcomes will be calculated using multivariate modeling, conducted at the Institute for Medical Informatics, Biometry and Epidemiology, Ludwig-Maximilians-University Munich, Germany.

Retrospective exploratory analysis can be performed by members of participating institutions of the registry upon application to the DGAV and approval by the StuDoQ Steering Committee, as detailed in the publication guidelines (available at www.dgav.de). Retrospective data provide a firm basis for the planning of prospective randomized clinical trials, especially with regard to sample size calculation and estimation of recruitment.

A further new field is registry-based randomized controlled trials (RRCTs). The TASTE trial (Thrombus Aspiration during ST-segment Elevation myocardial infarction) [20] can serve as an example: the investigators utilized the infrastructure of a population-based national registry, SCAAR (the Swedish Coronary Angiography and Angioplasty Registry). Using the existing registry infrastructure, the investigators were able to recruit and randomize over 6000 patients between June 2000 and September 2012 at an incremental cost of US$50 per patient.

#### Steering Committee

The Steering Committee is formed according to the registry constitution and consists of members of the DGAV StuDoQ|Pancreas development board, secretary and data safety commissioner of the DGAV, the CALGP, the participating institutions, the StuDoQ Registry management, as well as patient organizations.

### Domain: Standardization

#### Definitions and standard operating procedures

Where possible, international consensus definitions are used for the assessment of risk factors and outcome in pancreatic surgery. Important examples include the International Study Group of Pancreatic Surgery (ISGPS) definitions of postoperative pancreatic fistula (POPF) [21], delayed gastric emptying (DGE) [22], postpancreatectomy hemorrhage (PPH) [23], and the Center for Disease Control definition of surgical site infection (SSI) [24]. Standardized operating procedures (SOPs) are established for the internal automated plausibility check of data entry and query generation, the annual recertification of pancreatic cancer centers accredited by the German Cancer Society, the annual recertification of centers for pancreatic surgery accredited by the DGAV, and the annual institutional quality reports generated from the StuDoQ Registry.

#### Training for data entry and verification

Regular training courses for medical documenters/study nurses are offered by the DGAV. As most institutions already have established documentation by experienced personnel, and genuine interest in qualified documentation can be expected for certification purpose, participation in these courses is not considered mandatory. Two members of the Steering Committee are responsible to answer questions arising during data entry at the participating institutions.

### Domain: Validity of the sampling procedure

#### Definition of inclusion and exclusion criteria

Inclusion and exclusion criteria are defined as follows: included are all patients with informed consent and procedures involving pancreatic resection (total pancreatectomy, pancreatic head resection, distal pancreatectomy, central pancreatectomy, enucleation, drainage procedures, pseudocystostomy), bypass procedures for pancreatic or periampullary tumors or chronic pancreatitis (gastroenterostomy, hepatico- or cholecystoenterostomy) and exploratory laparoscopy or laparotomy for pancreatic or periampullary tumors. Periampullary tumors include pancreatic, distal bile duct, ampullary, and duodenal tumors. Exclusion criteria are lack of patient consent and procedures for acute necrotizing pancreatitis. Acute necrotizing pancreatitis, especially when surgery is required for infected necrosis or secondary complications, is a severe condition with a very high mortality. Pancreatic necrosectomy is hard to be controlled because it is rarely needed, performed in the emergency setting, not standardized and has heterogeneous outcome [25]. The DGAV, therefore, decided not to include necrosectomy in the StuDoQ Registry. This principle is also being followed by the German Initiative for Quality in Medical Care (Initiative Qualitätsmedizin, IQM) [26].

#### Completeness and representativity of data

By the defined inclusion and exclusion criteria, the whole spectrum of pancreatic surgery, except necrosectomy for acute necrotizing pancreatitis, is covered by the registry. In addition, the registry also encompasses palliative procedures for pancreatic and periampullary tumors. Complete documentation of all cases per participating center is a prerequisite for the annual recertification of centers for pancreatic surgery accredited by the DGAV. The registry can, therefore, deliver representative data on pancreatic surgery and surgery for periampullary tumors, with the exception of surgery for acute necrotizing pancreatitis.

On the basis of current routine administrative data [27], it is estimated that at present 10–20% of all pancreatic resections in Germany are documented in StuDoQ|Pancreas, with a strongly increasing tendency. Coverage of 10–20% might not be interpreted as representative. Participation in the registry has become mandatory since 2017 for institutions that aim to meet certification standards of the DGAV; therefore, numbers and coverage is expected to increase. On the other hand, 20% coverage is regarded as representative, for instance in the National Inpatient Sample of the USA, which covers 20% of hospital admissions [28, 29]. With the high case number covered per year, we are inclined to regard 20% also as representative.

Recent investigations show that 35% of pancreatic resections in Germany are performed in institutions with less than 10 cases per year (source: Federal Statistics Office of Germany), despite a national minimum case load policy of 10 cases per year [30]. An overall mortality of 10% for pancreatic resections in Germany is reported [15]. Perioperative mortality within the StuDoQ Registry currently meets an acceptable range of 2–6% (95% confidence interval, past 3 years). The DGAV endorses a minimum caseload of 30 pancreatic resections per year for certification [31]. The median annual caseload in the participating institutions over the past 3 years is in the range of 26–32 pancreatic resections per year.

In summary, the registry data seem representative for academic and nonacademic institutions with a medium to high caseload and acceptable morbidity and mortality, but obviously does not picture pancreatic surgery in the low-volume setting. As abundant international, as well as some national, data demonstrate that the latter is not desirable, the DGAV encourages centralization of pancreatic surgery in a minimum caseload policy. Underrepresentation of data from low-volume institutions in the registry is, therefore, not regarded as major drawback.

#### Consideration of sample size and effect

With respect to the various aims of the registry, sample size has to be considered. For the purpose of quality control by means of guideline adherence, reference values for quality indicators have been defined by the German Cancer Society, and sample size calculation seems not necessary for these purely descriptive statistics. In the setting of retrospective explorative data analysis, post-hoc power calculation can be performed if necessary. With regard to possible randomized registry trials, the registry provides several advantages: the registry infrastructure and data can be used for retrospective sample size calculation, prospective data collection, query generation, and data management. As the software and data are hosted by the DGAV, additional (and conditional) parameters can be added to the registry at any time.

#### Consideration of confounders and biases

Known risk factors causing confounding effects, like pancreatic texture or institutional and individual surgeon caseload are included in the registry baseline data, enabling risk adjustment of outcome measures. For a list of these risk factors see Additional file 1.

Regarding the intended use of the registry data, several sources of bias are possible, with the following methods to minimize bias: attrition bias is minimized by the requirement of complete documentation for certification purposes: to enforce completeness of data at the institutional level, external verification of total patient numbers by the institutional medicine controlling is required to obtain the annual institutional StuDoQ|Pancreas Report. Full documentation and verification is also required for annual institutional recertification by the DGAV. Detection bias is minimized by employment of international standard definitions for the outcome parameters (see above). The informed consent is usually obtained before surgery, but can also be obtained later in the process. Theoretically, there can be bias when mortality occurs before consent can be obtained or when patients decline consent. However, overall, the effect of this possible bias is considered minimal.

Retrospective comparative analysis of innovative surgical procedures may be prone to selection bias, a typical example being minimally invasive procedures. Appropriate case matching is a means to avoid this bias, but depends on sufficient numbers of suitable control cases [32, 33]. A large multicenter registry like StuDoQ|Pancreas provides an optimal means to achieve this. Reporting bias arises when negative or unexpected results are excluded from publication. Therefore, the DGAV|StuDoQ guidelines forbid such exclusion.

### Domain: Validity of data collection

#### Assessment of data: completeness, plausibility, distribution, and concordance

After browser-based data entry, the data are automatically checked for completeness and internal plausibility by internal rules. Completeness of major quality indicators is mandatory for inclusion in the annual institutional reports and certification purposes (for a list see Additional file 1). Missing or implausible items generate warning messages and queries. The data are usually entered after patient discharge. For generation of the annual institutional quality report, completeness of mandatory items is required.

#### Handling of missing data, follow-up, and dropout

The main emphasis of the registry is on perioperative outcome. Dropout during this relatively short time is minimal. Due to automatic plausibility checking and query generation during data entry, missing data for perioperative assessment have proven to be low. However, patient follow-up after discharge, including readmission, adjuvant and palliative therapies as well as survival, can also be documented. This feature constitutes a major advantage over routine administrative data, where readmissions are treated as new cases without connection to the primary case. It is also expected to be attractive, as patient follow-up is required for cancer center certification by the German Cancer Association (DKG). Handling of dropouts and missing data has to be decided depending on the intended analysis. Multiple imputation methods are suggested as methods of first choice [34].

#### Unique identifiers and risk of duplicate data entry

Data are entered in the registry in pseudonymized form at the institutional level without an over-institutional identifier. This precludes cross-validation with other over-institutional databases. Furthermore, there is no unified national pathology or cancer database that could serve this aim. As on the institutional level, cross-validation with medicine-controlling data is already in place, cross-validation by institutional pathology databases is not felt to add significant benefit. Efforts are under way to establish unique identifiers on the national level. Currently, there remains a theoretical possibility of duplicate data entry at different institutions. Nevertheless, repeated pancreatic surgery at different centers can be considered a very rare event.

#### Monitoring and audits

There is currently no complete external monitoring for the StuDoQ|Pancreas Registry. External monitoring at the institutional level is performed as annual audits by external auditors appointed by DGAV, which include data verification of random samples. Annual quality reports including data from the whole registry are issued to each participating institution and provided to the auditors, facilitating the detection of data-entry errors and deviations from average. Total case number and in-hospital mortality are verified annually by mandatory cross-validation with routine data (medical controlling) at the institutional level.

While extended monitoring in the sense of complete dataset verification would be desirable, it is outside the financial scope of the participating institutions and the DGAV: currently over 60 institutions and 1500–2000 cases per year would require a tremendous effort of personnel and cost. In view of possible future randomized registry trials, external monitoring will be needed for trial purposes.

### Domain: Validity of statistical analyses and reports

The aims and possible statistical analyses of the StuDoQ|Pancreas Registry are manifold. The following section incorporates aspects of the existing registry guidelines and gives further recommendations where appropriate.

#### Patient/data flow scheme

It is recommended that analyses are accompanied by a patient/data flow scheme as outlined in the Consolidated Standards of Reporting Trials (CONSORT) Statement [35].

#### Handling of missing data

Random missing data can be expected to be a frequent issue in the analysis of registry data. Preliminary results from the StuDoQ|Pancreas Registry demonstrate a low rate of missing data. It is recommended that either cases with missing data are excluded from analysis, or appropriate methods for imputation (for example, multiple imputation approaches) be applied [34]. When cases are excluded from analysis, this must be clearly stated; for example, by means of the patient/data flow scheme. Imputation methods should be given preference if significant bias can be expected by exclusion of cases.

#### Assessment of baseline data and outcome measures and evaluation of balance in comparative analyses

StuDoQ|Pancreas incorporates a broad spectrum of baseline parameters like demographic data, comorbidities, laboratory baseline data, and known risk factors (for details see Additional file 1). These can be used to evaluate for balance between groups in comparative analyses. Furthermore, these parameters provide a solid base for case-matching procedures. Regarding outcome measures, a high degree of standardization is achieved by incorporation of international standard definitions, as already reported in detail above.

#### Assessment of precision measures

There are no precision measures in the scope of the StuDoQ|Pancreas Registry.

#### Methods against bias and confounders

Possible biases and countermeasures have already been discussed above. The broad assessment of baseline parameters and risk factors detailed above allows for sophisticated methods to control confounders by case matching or multivariate modeling. Professional consultation and support regarding complex statistical issues regarding the StuDoQ Registry have been, and are, provided by the Institute for Medical Informatics, Biometry and Epidemiology, Ludwig-Maximilians-University Munich, Germany.

#### Adjustment for multiple inference testing

Where multiple inference testing is performed, adjustment is strongly suggested. The applications for analyzing data from the registry are checked for this type of bias. In the setting of prospective studies (like RRTs or observational studies), methods of adjustment have to be defined in the trial protocol.

#### Assessment of relative and absolute effects, adjusted and unadjusted results

Studied effects (like outcome measures or quality indicators) should be reported as relative and absolute numbers, and be adjusted for confounders if appropriate.

#### Multivariate modeling for complex questions

By the many baseline, operative, outcome, and follow-up parameters, the StuDoQ|Pancreas Registry provides a broad basis for sophisticated multivariate modeling. Investigators are encouraged to take advantage of this, and professional consultation and support are provided by the Institute for Medical Informatics, Biometry and Epidemiology, Ludwig-Maximilians-University Munich, Germany.

#### Consideration of timeline in longitudinal data

StuDoQ|Pancreas is not primarily designed as a longitudinal registry. However, documentation of detailed follow-up, mostly regarding oncologic aspects, is possible. Follow-up is further mandatory for pancreatic cancer patients for certification of pancreatic cancer centers by the DKG. Therefore, it is possible that treatment effects be assessed longitudinally, especially in oncologic patients.

#### Control of cluster effects

StuDoQ is a multicenter registry; therefore, cluster effects can arise with regard to differing treatment standards in the participating centers. One important aspect in pancreatic surgery is the strong volume-outcome relationship demonstrated in large-scale multicenter studies and insurance-based routine data. The data structure of StuDoQ|Pancreas allows for assessment of cluster effects, by institution as well as by surgeon, in a pseudonymized form.

#### Validity of statistical analyses and reports

As outlined in this section, StuDoQ|Pancreas provides the basis for powerful statistical analysis of baseline parameters, risk factors, perioperative outcome as well as follow-up in oncologic patients. Before publication, all reports have to be checked by the Steering Committee.

### Domain: General demands for registry quality

#### Transparency towards limitations

There are several limitations of the StuDoQ|Pancreas Registry which are treated with a high level of transparency: While data acquisition is prospective, quality analyses other than possible RRTs will be of retrospective nature. There is no unique identifier for patients, leaving a possibility of duplicate data entry at distinct institutions. Furthermore, there is at present no external monitoring, but annual external audits with limited internal cross-validation and random sample validation for certification purposes have been established. In addition, selection bias cannot yet be ruled out.

#### Acceptance among patients and institutions

With growing political interest for quality management in health care in Germany, it is expected that the StudoQ|Pancreas Registry will be recognized by surgeons as a valuable tool to assess institutional quality and to obtain institutional certification by the DGAV. Due to promotion through the DGAV and at several national scientific meetings and congresses, the StuDoQ|Pancreas registry has received significant acceptance. At the time of writing, over 50 institutions, from academic centers to community hospitals, participate in the registry. An annual case number of over 1000 cases are expected for 2016, accounting for an estimated 10–20% of all pancreatic surgery procedures in Germany, further increasing a current dataset of over 4000 cases since 2013. In summary, acceptance can be considered high, and further expansion is to be expected.

#### Transparency and scientific independence

The constitution of the DGAV StuDoQ Registry, including data protection and publication guidelines, are transparent to the surgical community as well as to the public and can be accessed online through the DGAV Internet website (www.dgav.de).

#### Flexibility and adaptability

By implementation as a nonproprietary code in Python (www.python.org) and R software (www.r-project.org), the registry software retains maximal flexibility. In addition, personnel for IT support, maintenance, and software development, as well as user feedback management, are part of the DGAV. This ensures the possibility of analysis and adjustments at any time. Of note, browser-based data export at the institutional level is possible. This feature provides the participating institutions with a powerful tool to perform independent quality assessment or scientific studies at the institutional level. Thereby, at the same time, the registry sets a national standard for pancreatic surgery documentation and research.

#### Topicality

The registry enables the surgeons and institutions of Germany to participate in some of the most relevant topics in current health care and surgery, like routine and scientific evaluation of quality indicators, assessment of effectiveness of surgical interventions and design of randomized registry trials.

## Conclusion

Inspired by current political and professional debate, the German Society for General and Visceral Surgery (DGAV) established StuDoQ|Pancreas, a prospectively maintained registry for pancreatic surgery. The aims of this registry are to enable the surgical community to assess quality and effectiveness of pancreatic surgery and surgical interventions, to provide a framework for center certification and RRCTs. By its organization structure, flexible technical implementation and public transparency, the registry sets a new standard for pancreatic surgery documentation and research in Germany.

## Additional file

Additional file 1: Table S1. Parameters assessed in the DGAV StuDoQ|Pancreas Registry. (DOCX 56 kb)

## Abbreviations

ACS-NSQIP: American College of Surgeons National Surgical Quality Improvement Program
CALGP: German Chapter of the International Hepato-pancreato-biliary Association
DGAV: Deutsche Gesellschaft für Allgemein- und Viszeralchirurgie/German Society for General and Visceral Surgery
DGE: Delayed gastric emptying
DKG: German Cancer Association
DNVF: German Network Health Services Research
DRG: Diagnostic-related categories
eCRF: Electronic Case Report Form
IHPBA: International Hepato-pancreato-biliary Association
ISGPS: International Study Group of Pancreatic Surgery
POPF: Postoperative pancreatic fistula
PPH: Postpancreatectomy hemorrhage
RCTs: Randomized controlled trials
RRCTs: Registry-based randomized controlled trials
SOPs: Standardized operating procedures
