# Supplementary material for: The pancreatic surgery registry (StuDoQ|Pancreas) of the German Society for General and Visceral Surgery (DGAV) – presentation and systematic quality evaluation
Source: Trials. 2017 Apr 5;18:163. doi: 10.1186/s13063-017-1911-x (PMC5382382; doi:10.1186/s13063-017-1911-x)
Supplement: Additional file 1: Table S1. — Parameters assessed in the DGAV StuDoQ|Pancreas Registry. (DOCX 56 kb) [file 13063_2017_1911_MOESM1_ESM.docx]

**Table S1. Parameters assessed in the DGAV-StuDoQ|Pancreas registry.** Known risk factors in pancreatic surgery (RF) and parameters assessed according to ISGPS definitions are marked in the respective column. Abbreviations: RF known risk factor in pancreatic surgery, ISGPS international study group for pancreatic surgery, ASA American Society of Anaesthesiolologists, NYHA New York Heart Association, COPD chronic obstructive pulmonary disease, PAOD periphereal arterial occlusive disease, EUS endoscopic ultrasound, ERCP endoscopic retrograde cholangio-pancreatography, MRI magnetic resonance imaging, CT computer aided tomography, MPD main pancreatic duct, LAD lymphadenectomy, PVR mesenterico-portal resection, POPF postoperative pancreatic fistula, PPH postpancreatectomy hemorrhage, DGE delayed gastric emptying, SSI surgical site infection, preOP preoperative, postOP postoperative.

| **category** | **variable** | **RF** | **ISGPS** |
| --- | --- | --- | --- |
| baseline parameters | sex | X |  |
|  | age | X |  |
|  | body mass index | X |  |
|  | elective or emergency surgery | X |  |
|  | ASA category | X |  |
|  | need of care | X |  |
|  | endoscopic retrograde drainage | X |  |
|  | chemo or radiotherapy |  |  |
|  | weight loss | X |  |
|  | hospital stay |  |  |
| symptoms | symptom: emesis |  |  |
|  | symptom: hypoglycemia |  |  |
|  | symptom: nausea |  |  |
|  | symptom: pain |  |  |
|  | symptome: jaundice |  |  |
| comorbidity | alcohol abuse |  |  |
|  | arterial hypertension with medication |  |  |
|  | anticoagulant medication |  |  |
|  | cerebrovascular event |  |  |
|  | coronary artery disease |  |  |
|  | corticosteroid medication |  |  |
|  | diabetes mellitus | X |  |
|  | disseminated malignancy |  |  |
|  | hemodialysis |  |  |
|  | history of acute pancreatitis | X |  |
|  | history of chronic pancreatitis | X |  |
|  | immunosuppressive medication |  |  |
|  | liver cirrhosis | X |  |
|  | esophageal varices |  |  |
|  | ascites |  |  |
|  | NYHA stage |  |  |
|  | sepsis |  |  |
|  | severe COPD |  |  |
|  | severe PAOD |  |  |
|  | smoking pack years | X |  |
|  | smoking until [years preoperative] |  |  |
| preoperative diagnostics | ultrasonography |  |  |
|  | EUS |  |  |
|  | CT |  |  |
|  | MRI |  |  |
|  | ERCP |  |  |
|  | ERCP complications |  |  |
|  | EUS complications |  |  |
|  | cT category |  |  |
|  | cN category |  |  |
|  | cM: liver metastasis |  |  |
|  | cM: other metastasis |  |  |
|  | cM: pulmonary metastasis |  |  |
|  | histology / cytology |  |  |
|  | histologic / cytologic diagnosis |  |  |
|  | tumor localization |  |  |
|  | diagnosis |  |  |
| preoperative laboratory | CA 19.9 |  |  |
|  | CEA |  |  |
|  | hemoglobin |  |  |
|  | leucocytes |  |  |
|  | creatinine | X |  |
|  | amylase |  |  |
|  | lipase |  |  |
|  | AP |  |  |
|  | GGT |  |  |
|  | bilirubin | X |  |
|  | albumin | X |  |
|  | C-reactive protein | X |  |
| operation details | operation type |  | X |
|  | operation time | X |  |
|  | minimal invasive technique |  |  |
|  | conversion cause |  |  |
|  | time to conversion |  |  |
|  | pancreatic anastomosis / closure method |  |  |
|  | pancreatic texture (soft / hard) | X |  |
|  | MPD diameter (normal / dilated) | X |  |
|  | MPD stent placement |  |  |
|  | pancreatic transsection method |  |  |
|  | resectate length |  |  |
|  | distance of transsection from mesenteric root |  |  |
|  | LAD (standard / extended) | X | X |
|  | PVR | X |  |
|  | arterial resection | X |  |
|  | splenectomy |  |  |
|  | spleen preservation planned |  |  |
|  | spleen preserved |  |  |
|  | spleen preservation technique |  |  |
|  | spleen injury |  |  |
|  | colon resection | X |  |
|  | gastric resection |  |  |
|  | intestinal resection |  |  |
|  | kidney resection |  |  |
|  | liver resection | X |  |
|  | somatostatin analogue application |  |  |
|  | red blood cell transfusion | X |  |
|  | peridural analgesia |  |  |
|  | thromboprophylaxsis |  |  |
|  | antibiotic prophylaxsis |  |  |
| perioperative outcome | Clavien-Dindo category |  |  |
|  | reoperation |  |  |
|  | POPF |  | X |
|  | PPH |  | X |
|  | DGE |  | X |
|  | hepaticoenterostomy leak |  |  |
|  | gastroenterostomy leak |  |  |
|  | intraabdominal abscess |  |  |
|  | SSI |  |  |
|  | burst abdomen |  |  |
|  | mechanical ileus |  |  |
|  | other surgical complication |  |  |
|  | acute myocardial infarction |  |  |
|  | pneumonia |  |  |
|  | unplanned ventilation |  |  |
|  | diabetes mellitus |  |  |
|  | hemodialysis |  |  |
|  | oral enzyme supplementation |  |  |
|  | stroke |  |  |
|  | thromboembolism |  |  |
|  | other nonsurgical complication |  |  |
|  | mortality cause |  |  |
|  | ICU stay [days] |  |  |
|  | overallall hospital stay [days] |  |  |
|  | discharge destination |  |  |
|  | readmission |  |  |
| histology | histology diagnosis |  |  |
|  | pT category |  |  |
|  | pN category |  |  |
|  | pM category |  |  |
|  | tumor grade (G) |  |  |
|  | positive lymph nodes |  |  |
|  | total lymph node number |  |  |
|  | CRM workup |  |  |
|  | margin status |  |  |
|  | margin status anterior |  |  |
|  | margin status posterior |  |  |
|  | margin status medial / mesopancreas |  |  |
|  | margin status pancreas parenchyma |  |  |
|  | margin status bile duct |  |  |
|  | margin status duodenum |  |  |
|  | lymphangiosis |  |  |
|  | hemangiosis |  |  |
|  | perineural infiltration |  |  |
| oncologic therapy | preOP tumor board consultation |  |  |
|  | preOP tumor board recommendation |  |  |
|  | therapy guideline adherence |  |  |
|  | preOP therapy intent |  |  |
|  | preOP Cx recommended |  |  |
|  | preOP Rx recommended |  |  |
|  | preOP Cx end cause |  |  |
|  | preOP Cx cancellation cause |  |  |
|  | preOP Rx end cause |  |  |
|  | preOP Rx cancel cause |  |  |
|  | best supportive care |  |  |
|  | postOP tumor board consultation |  |  |
|  | postOP tumor board recommendation |  |  |
|  | postOP therapy intent |  |  |
|  | postOP therapy recommendation |  |  |
|  | postOP Cx recommended |  |  |
|  | postOP Rx recommended |  |  |
|  | postOP Cx end cause |  |  |
|  | postOP Cx cancellation cause |  |  |
|  | postOP Rx end cause |  |  |
|  | postOP Rx cancellation cause |  |  |
|  | psychooncologic therapy |  |  |
|  | social service consultation |  |  |
|  | doctors consultation at discharge |  |  |
|  | presentation in MM conference |  |  |
| follow-up | vital status |  |  |
|  | death cause |  |  |
|  | conducted therapy |  |  |
|  | local recurrence |  |  |
|  | new liver metastasis |  |  |
|  | new lung metastasis |  |  |
|  | new other metastasis |  |  |
|  | other neoplasia |  |  |
|  | local recurrence resection |  |  |
|  | liver metastasis resection |  |  |
|  | lung metastasis resection |  |  |
|  | other metastasis resection |  |  |
| Institutional factors | institutional caseload | X |  |
|  | surgeon caseload | X |  |
